# Nonadiabatic Quantum Dynamics of Molecules Scattering from Metal Surfaces

**DOI:** 10.1021/acs.jctc.4c01586

**Published:** 2025-01-28

**Authors:** Riley J. Preston, Yaling Ke, Samuel L. Rudge, Nils Hertl, Raffaele Borrelli, Reinhard J. Maurer, Michael Thoss

**Affiliations:** † Institute of Physics, University of Freiburg, Hermann-Herder-Strasse 3, 79104 Freiburg, Germany; ‡ Department of Chemistry and Applied Biosciences, ETH Zürich, 8093 Zürich, Switzerland; § Department of Chemistry, University of Warwick, Gibbet Hill Road, Coventry CV4 7AL, U.K.; ∥ Department of Physics, University of Warwick, Gibbet Hill Road, Coventry CV4 7AL, U.K.; ⊥ DISAFA, University of Torino, I-10095 Grugliasco, Italy

## Abstract

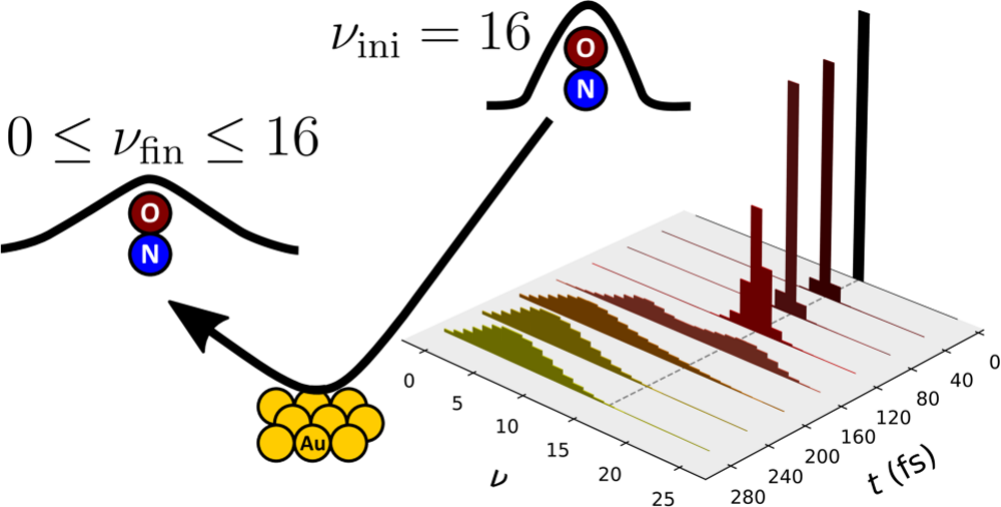

Nonadiabatic coupling between electrons and molecular motion at metal surfaces leads to energy dissipation and dynamic steering effects during chemical surface dynamics. We present a theoretical approach to the scattering of molecules from metal surfaces that incorporates all nonadiabatic and quantum nuclear effects due to the coupling of the molecular degrees of freedom to the electrons in the metal. This is achieved with the hierarchical equations of motion (HEOM) approach, combined with a matrix product state representation in twin space. The method is applied to the scattering of nitric oxide from Au(111), for which strongly nonadiabatic energy loss during scattering has been experimentally observed, thus presenting a significant theoretical challenge. Since the HEOM approach treats the molecule–surface coupling exactly, it captures the interplay between nonadiabatic and quantum nuclear effects. Finally, the data obtained by the HEOM approach are used as a rigorous benchmark to assess various mixed quantum-classical methods, from which we derive insights into the mechanisms of energy dissipation and the suitable working regimes of each method.

## 1 Introduction

Understanding the dynamics of molecules interacting with metal surfaces is vital in a wide range of scenarios, such as reactive and catalytic processes,^1−3^ charge transport through molecular nanojunctions,^4−7^ and scattering experiments.^8−11^ Often critical in these setups is the influence of the metal electrons on the dynamics of the molecule, in particular due to nonadiabatic processes that lead to the creation of electron–hole pair (EHP) excitations within the metal. For example, nonadiabatic processes lead to vibrationally inelastic scattering,^12^ chemically induced currents,^13^ dynamical steering effects,^14^ and ultrashort vibrational lifetimes.^15−17^ Consequently, theoretical treatments of such situations require sophisticated methods capable of going beyond the Born–Oppenheimer approximation.^18^

The scattering of molecules from metal surfaces is a prototypical example of surface dynamics and has motivated the development of a plethora of nonadiabatic theoretical methods.^9^ These range from mixed quantum-classical (MQC) approaches that treat the motion of atomic nuclei classically while being coupled to quantum electronic degrees of freedom, to fully quantum approaches. To the first category belong methods such as Ehrenfest dynamics,^19−21^ electronic friction approaches,^22−27^ and surface-hopping techniques.^28−30^ Fully quantum treatments, in contrast, are able to propagate the many-dimensional nuclear wavepacket but generally rely on an approximate treatment of the coupling to surface electrons^31−33^ or neglect the role of electronic excitations in the metal entirely.^34−36^

While these methods have had impressive success for a multitude of scattering problems, they often fail to describe the full complexity of the dynamics. For instance, one of the most well-explored scattering experiments for probing nonadiabatic effects is that of a NO molecule scattering from Au(111). Here, it has been shown that the coupling to EHPs in the surface strongly impacts the dynamics of the scattering molecule,^37−39^ leading to multiquantum relaxation in the vibrational state of the molecular bond.^40^ However, theoretical treatments of NO scattering are often unable to accurately capture the dynamics observed in experiments, usually underpredicting the amount of vibrational relaxation.^40^ The origins of this failure are hard to diagnose due to the combination of different sources of errors in the description of the electronic structure or the coupled electron–nuclear dynamics of realistic systems. This problem can only be tackled by separately benchmarking the quality of electronic structure predictions^41^ and by quantifying the basic ability of the employed nonadiabatic dynamics method to capture all relevant dynamic effects. Unfortunately, existing MQC methods vary widely in their quantitative predictions.^42^

This is the primary motivation for this work, wherein we employ a fully quantum mechanical approach for simulating the scattering of molecules from metal surfaces that incorporates the molecule–surface interaction exactly. This is achieved via the hierarchical equations of motion (HEOM) method, which is a numerically exact technique for modeling the dynamics of open quantum systems.^43^ While HEOM has previously been applied to molecular bond rupture at surfaces,^7,44,45^ nonequilibrium chemical reactions,^46^ desorption from surfaces,^47^ and the scattering of a single atom from a surface,^48^ in this work, we present the first application of HEOM to the scattering of a molecule with multiple nuclear degrees of freedom from a metal surface. After establishing the general theoretical framework, we exemplify the approach via application to the longstanding problem of NO scattering from Au(111), where previous quantum calculations have relied on an approximate description of the coupling to the surface electrons.^33^ We observe multiquantum relaxation of the bond vibrational state due to coupling to EHPs in the surface, in accordance with experiment. In doing so, we demonstrate that the HEOM approach not only represents a significant step forward in the modeling of nonadiabatic scattering of molecules from metal surfaces but also that it can be further applied as a benchmark both for models and other approximate theoretical methods.

## 2 Theory

Throughout this work, we used natural units such that *ℏ* = *e* = *m*_e_ = 1. We employ a Newns–Anderson Hamiltonian, which is the standard model for dynamics at metal surfaces.^49,50^ The Hamiltonian is partitioned according to
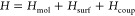
1where *H*_mol_ corresponds to the scattering molecule, *H*_surf_ to the surface, and *H*_coup_ to the coupling between the molecule and surface. The molecular Hamiltonian is given by
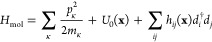
2where **x** is the vector of nuclear coordinate operators, whose elements are indexed by κ, with reduced mass *m*_κ_, and *p*_κ_ is the set of corresponding conjugate momenta. The vibrational potential energy surface for the neutral molecule is given by *U*_0_(**x**). The *d*_*i*_^†^/*d*_*i*_ are the creation/annihilation operators for the electronic state *i* on the molecule, where the energies and interstate coupling strengths are contained within *h*_*ij*_. The electrons in the metal are modeled as a noninteracting reservoir of electrons such that
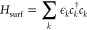
3where *c*_*k*_^†^/*c*_*k*_ denote the creation/annihilation operators for the state *k* with energy ϵ_*k*_. The surface is held at local equilibrium such that the occupation function for electrons and holes is
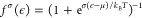
4where σ ∈ {+, −}, with σ = + for electrons and σ = – for holes, while *T* is the temperature, and μ is the chemical potential. The electronic coupling between the molecule and the surface is given by
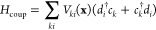
5with coordinate-dependent coupling elements *V*_*ki*_ (**x**).

After integrating out the surface degrees of freedom, the influence of the molecule–surface coupling on the dynamics of the molecule arises via the two-time bath correlation functions,
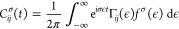
6where Γ_*ij*_(ϵ) is the spectral density function, defined as
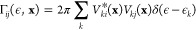
7The energy dependence of the spectral density is assumed to take the form of a Lorentzian such that
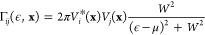
8where *W* is the bandwidth, and the strength of the electronic coupling to the surface is described by *V*_*i*_. A fundamental step in the HEOM approach is to decompose the bath correlation functions in eq 6 as a power series of exponential functions. For finite temperature, it can be accurately approximated with only a small, finite number of poles, *P*, such that
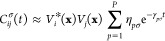
9In this work, we employ the barycentric sum-over-pole decomposition scheme to calculate η_*p*σ_ and γ_*p*σ_, where each depends on the temperature of the surface, as detailed in previous studies.^51,52^ The decomposition in eq 9 can be interpreted as a mapping of the continuum of electronic states in the metal onto a finite set of *K* = 2*N*_e_*P* effective virtual Fermionic bath states, where *N*_e_ is the number of electronic degrees of freedom of the molecule.

The dynamics of the system can then be expressed as a hierarchy of equations of motion^43,53−62^
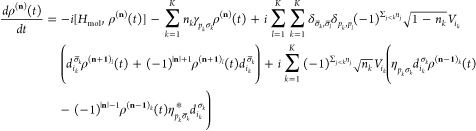
10Here, **n** = [*n*_1_, *n*_2_,···,*n*_*K*_], where *n*_*m*_ is the occupation of the *m*^th^ virtual bath state, which can be either 0 or 1. The norm is  and σ̅ = −σ. The creation and annihilation operators have also been rewritten in this notation such that *d*_*i*_*k*__^+^ = *d*_*i*_*k*__^†^ and *d*_*i*_*k*__^–^ = *d*_*i*_*k*__. The reduced density matrix for the scattering molecule is obtained when all virtual bath states are unoccupied such that ρ_mol_ = ρ^([0,0,···,0])^. Each subsequent configuration of bath occupations, **n**, results in an additional auxiliary density operator (ADO), ρ^(**n**)^, which together collectively contain information about the influence of the bath on the dynamics of the molecule. The hierarchy is formed via the coupling of each ADO, ρ^(**n**)^, up/down to ADOs of a higher/lower tier, ρ^(**n±1**_*k*_)^, where
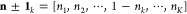
11

The direct propagation of eq 10 quickly becomes computationally infeasible for systems with a large dimensionality of the Hilbert space, limiting its application to scattering systems consisting either of only a single nuclear degree of freedom or restrictively small basis sets. Here, we overcome this limitation and enable the treatment of realistic molecular scattering systems consisting of multiple nuclear degrees of freedom, by reformulating the HEOM into a Schrödinger-like equation of motion for an extended wave function in twin space, which then facilitates the use of a matrix product state (MPS) representation of the state of the system.^52,63,64^ In doing so, the dimensionality of the molecular density matrix and ADOs scales polynomially with respect to the size of the vibrational basis set rather than exponentially, which is especially critical for the accurate treatment of vibrational, rotational, and translational degrees of freedom of a molecule, where a large basis set is often needed.

In the reformulation,^53^ the ADOs are recast as tensors in the twin space according to
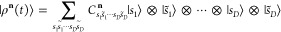
12where *D* is the number of degrees of freedom of the molecule. Subsequently, each operator for the molecule in eq 2 adopts two twin-space counterparts defined according to
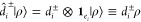
13
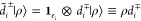
14
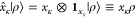
15
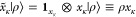
16The operators with a hat act on the physical degrees of freedom, while those with a tilde act on ancilla degrees of freedom. One can additionally define a set of ad hoc creation/annihilation operators that act on the Fock state for the virtual bath states, |**n**⟩ = |*n*_1_···*n*_*K*_⟩. These, along with an additional parity operator, *I*^>^, are defined as
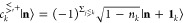
17
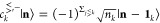
18
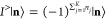
19The contribution of all ADOs is then contained within an extended pure-state wave function for the molecule and surface, given by
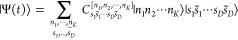
20It is defined such that any ADO can be readily extracted via a projection onto the relevant Fock state for the bath,
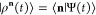
21The time evolution of the extended wave function follows
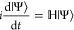
22where the super-Hamiltonian, , is
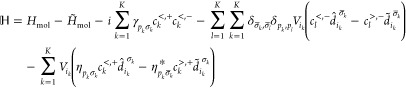
23The form of eq 22 is conducive to the use of tensor network schemes such as the matrix product state (MPS) formalism, which is employed here. In doing so, the high-rank coefficient tensor, *C*_*s*_1_*s̃*_1_···*s*_*D*_*s̃*_*D*__^[*n*_1_,*n*_2_,···,*n*_*K*_]^, is decomposed as a product of low-rank matrices
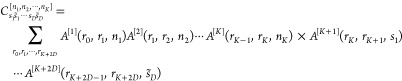
24where *A*^[*i*]^ is a rank-3 tensor with two virtual indices (*r*_*i*–1_, *r*_*i*_) and one physical index. To enforce that the decomposition for a given element of the tensor of coefficients evaluates to a scalar, an open boundary condition is applied where *r*_0_ = *r*_*K*+2*D*_ = 1. For an arbitrary state, the decomposition in eq 24 is formally exact in the limit of infinite bond dimension, *r*_*i*_. In practice, a truncation to a finite bond dimension is applied. The accuracy of the MPS representation of the state can then be tuned by manipulating the bond dimensions in the MPS. The super-Hamiltonian is analogously decomposed as a matrix product operator
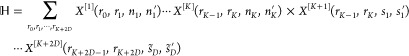
25

For the propagation of the MPS in eq 22, we employ the time-dependent alternating minimal energy (tAMEn) solver proposed by Dolgov^65^ and applied to HEOM by Borrelli et al.,^66,67^ wherein the MPS bond dimensions and time-step size are adaptively altered throughout the propagation according to a specified error tolerance. The results presented here are converged with respect to this error tolerance.

## 3 Results

### 3.1 Model

The utility of the introduced method is now demonstrated via its application to the modeling of NO/Au scattering. The model employed here is taken from ref. (42) and is parametrized for the scenario when the NO molecule is incident on the surface with the N atom facing down toward the surface.^68^ A reduced description of the NO molecule is considered in which only two nuclear degrees of freedom are retained; the center-of-mass distance from the surface, *z*, and the NO bond stretching, *r*. We additionally consider the molecule to have only a single electronic level that participates in the dynamics, which can either be occupied or unoccupied. The diabatic potential energy surfaces that describe the molecule are given by
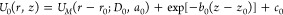
26
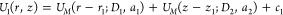
27where *U*_*M*_ is the Morse potential, defined as
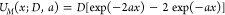
28The two diabatic states represent the neutral state, *U*_0_, and the negatively charged anionic state, *U*_1_, of the molecule. The parameters in eqs 26 and 27 were determined by parametrization against first-principles data based on constrained density functional theory,^68^ where the number of electrons in the molecule was constrained to a fixed value.

**Figure 1:**
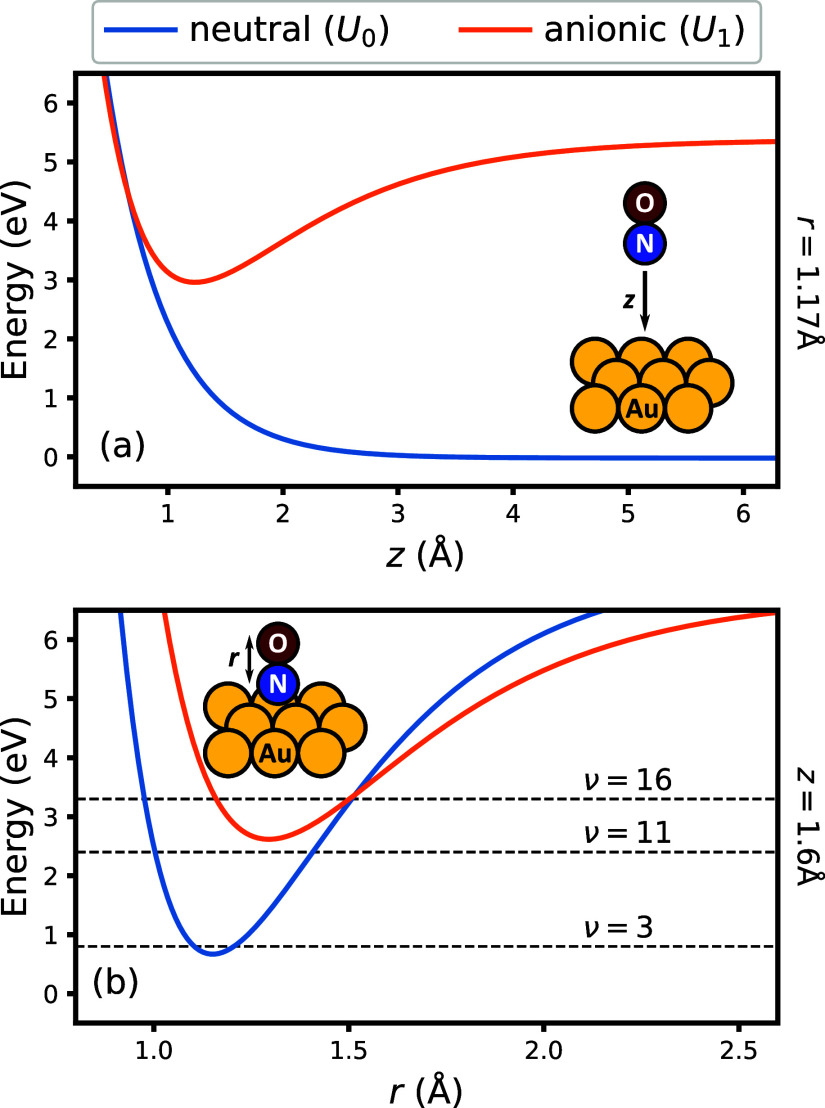
One-dimensional cuts of the neutral and charged potential energy surfaces. (a) As a function of *z* for *r* = 1.17 Å. (b) As a function of *r* for *z* = 1.6 Å, where the energies of the ν ∈ {3, 11, 16} states are also shown.

The molecular Hamiltonian is then given by
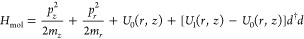
29The masses *m*_*z*_ and *m*_*r*_ correspond to the total mass of the NO molecule and the reduced mass for the bond, respectively. Example one-dimensional cuts along each axis of the potentials are shown in Figure 1, and these will act as a helpful guide for the discussions in the following sections. The electronic coupling strength is taken to be dependent only on *z*, with two parameters that have been chosen to best approximate the ground-state adiabatic potential energy surface for the NO/Au(111) system. It takes the form
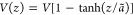
30The temperature of the surface is set to 300 K. The full set of model parameters can be found in Section 1 of the Supporting Information or in ref (42).

The initial wavepacket for the molecule is described by a product state, wherein the bond stretch mode is initialized in an eigenstate of *U*_0_ in the limit *z* → *∞*. The eigenstates are denoted by ν, while the initial state is ν_ini_. The *z*-component of the initial wavepacket is taken to be a Gaussian wavepacket incident on the surface, with an average kinetic energy, , as given by
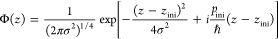
31The parameters in eq 31, along with all other associated parameters and details of the computation, are provided in Section 2 of the Supporting Information.

### 3.2 Energy Transfer Dynamics of Molecular Scattering

**Figure 2:**
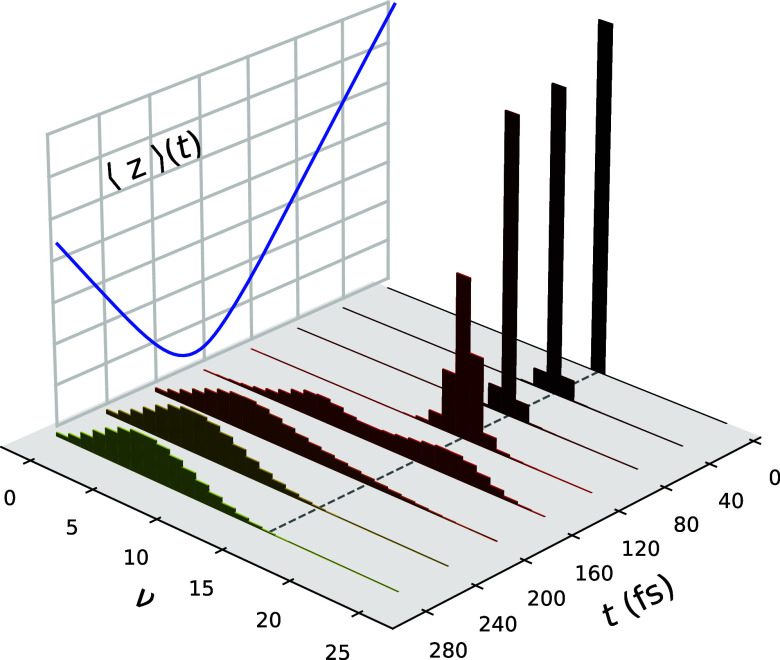
Time dependence of the vibrational state populations, *P*_ν_(*t*), with ν_ini_ = 16 and KE_ini_ = 1.0 eV. The background shows the time dependence of ⟨*z*⟩.

Here, we present exploratory results of HEOM simulations for the scattering process. As a first demonstration, we calculate the time-dependent populations of vibrational states of the bond stretching mode according to
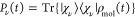
32where *P*_ν_ is the population of state ν, obtained via a projection onto the corresponding basis state |χ_ν_⟩. Figure 2 shows the time dependence of the population of bond vibrational states during the scattering process when initialized in the state ν_ini_ = 16 with an initial translational kinetic energy of KE_ini_ = 1.0 eV. In addition, the average distance from the surface ⟨*z*⟩(*t*) is depicted. On approach toward the surface, the bond experiences both relaxation and excitation from the initial state due to energy exchange with electrons in the surface. At the point of the closest approach, when ⟨*z*⟩ is at the minimum, the vibrational distribution is bimodal, where the occupation of higher vibrational states is also correlated with a higher electronic occupation of the molecule (see Figure S1 in the Supporting Information). Upon scattering away from the surface, the highly excited vibrational bond states relax to lower energy states, and the state of the bond converges to the final distribution.

**Figure 3:**
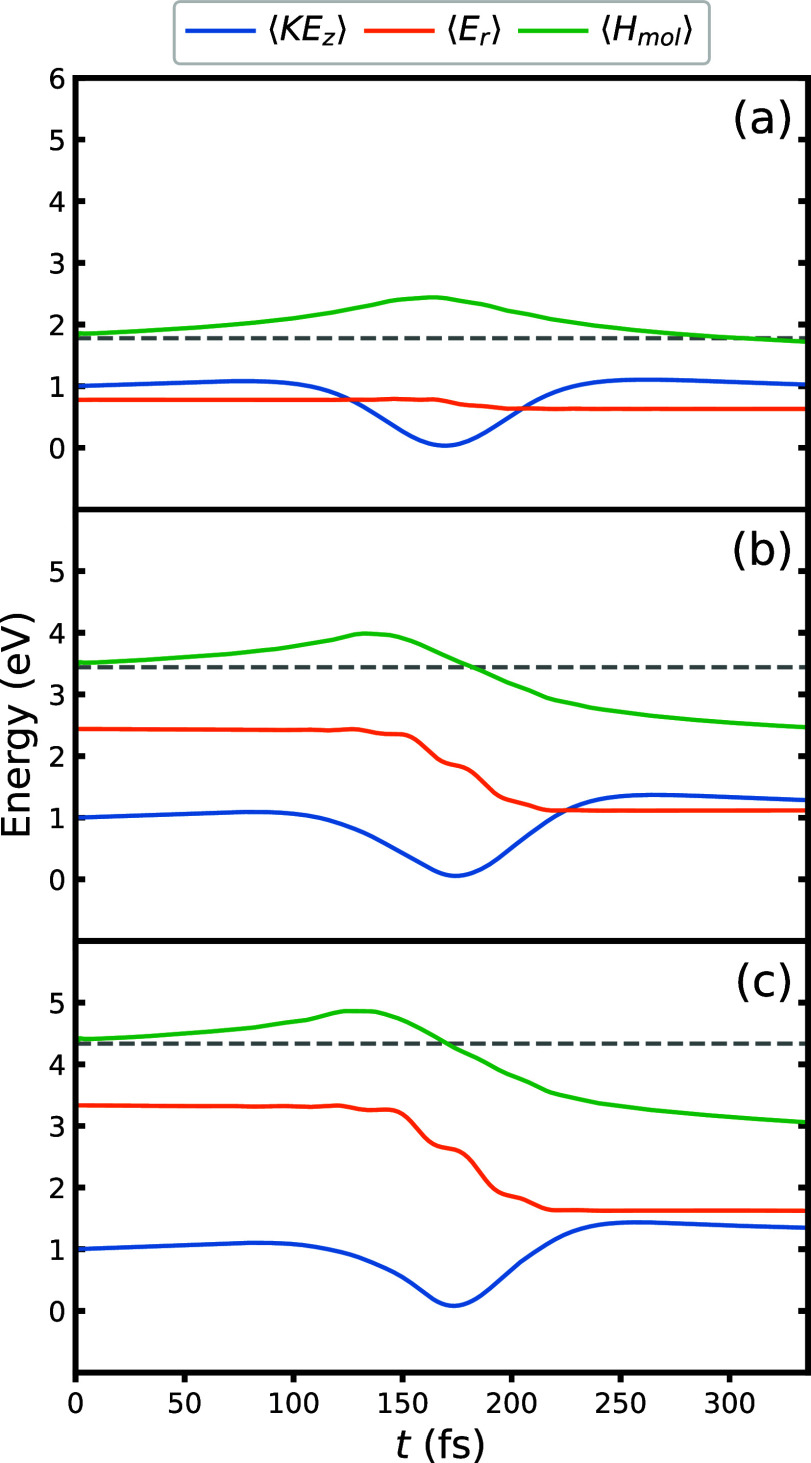
Time dependence of the mean molecular energy along with its components, the mean translational kinetic energy, and the energy stored in the molecular bond. KE_ini_ = 1.0 eV with (a) ν_ini_ = 3, (b) ν_ini_ = 11, and (c) ν_ini_ = 16. The dashed line serves as a reference for the initial ⟨*H*_mol_⟩.

It is insightful to analyze the time-dependent components of the energy in the system to better understand the dynamical processes at play. In Figure 3, the time dependence of the molecular energy, ⟨*H*_mol_⟩, along with the corresponding translational kinetic energy, ⟨KE_*z*_⟩, and bond energy, ⟨*E*_*r*_⟩ are shown for three different initial vibrational states, where KE_ini_ = 1.0 eV. The bond energy is defined as
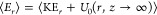
33In all cases, the loss of energy of the molecule arises almost entirely via the relaxation of the vibrational state of the bond. This energy is primarily lost to the surface in the form of electron–hole pair excitations. However, some of the energy is instead transferred to the translational kinetic energy of the molecule upon scattering. This is evidence for EHP-mediated weak translation–vibration coupling, which has been previously observed in NO scattering experiments.^37^ By virtue of considering only a 2D model, energy transfer to other nuclear degrees of freedom such as the molecule’s rotational motion is not considered.^14,31,37^

The loss of energy of the molecule to the surface electrons, calculated as the change in ⟨*H*_mol_⟩ over the scattering event, can be used to quantify the prevalence of nonadiabatic effects contributing to the dynamics since energy can only be lost to the surface via nonadiabatic EHP excitation effects. In the ν_ini_ = 3 case, the molecule loses 0.1 eV to the surface electrons throughout the scattering process, whereas a higher initial vibrational state yields a much larger energy loss: 1.0 eV for ν_ini_ = 11 and 1.3 eV for ν_ini_ = 16. This can be rationalized via reference to Figure 1b, where the energy of the ν = 16 state is near the energy of intersection between the neutral and charged potentials when the molecule is close to the surface. As a result, energy exchange processes between the molecule and the electrons in the surface are more accessible in comparison to when the molecule is initialized in a less excited state. When initialized in the ν_ini_ = 3 state, the molecule interacts comparatively weakly with the electronic states in the surface at all times throughout the scattering event.

The energy lost by the molecule is also strongly dependent on its incoming kinetic energy. As demonstrated in Table 1 for a molecule initialized in the ν_ini_ = 11 state, the energy loss of the molecule to the EHPs is small for KE_ini_ = 0.2 eV but becomes significantly larger as KE_ini_ increases. This behavior is clarified by Figure 1a, wherein a large kinetic energy is required to climb the potential barrier before being able to strongly interact with the electrons in the surface. Furthermore, the energy transfer from the bond degree of freedom to the translational motion of the molecule, as mediated by the electrons on the surface, becomes proportionally larger with increasing KE_ini_.

**Table 1:** Energy Loss Pathways for ν_ini_ = 11^a^

	KE_ini_ = 0.2 eV	KE_ini_ = 0.5 eV	KE_ini_ = 1.0 eV
energy lost from bond	0.17 eV	0.89 eV	1.32 eV
% lost to EHPs	55.3	77.3	73.9
% transferred to KE_*z*_	9.6	15.8	21.5

a The displayed percentages are in terms of the energy lost from the bond degree of freedom.

### 3.3 Final Vibrational State Distributions

In accordance with the bulk of previous literature for the system under consideration, we analyze the final vibrational state distributions of the bond stretching after scattering. Beyond providing insight into the role of EHPs in the relaxation of the bond stretch mode state, we additionally highlight the value of the HEOM approach as a benchmark reference technique. To accomplish this, the HEOM method is here compared against a range of methods that treat the vibrational degrees of freedom of the NO molecule classically for the same model system and the same physical parameters. Each MQC method considered incorporates the influence of nonadiabatic effects in the system via different assumptions. The comparison focuses on the final distribution of bond stretching state populations, which is also the main observable of interest.

**Figure 4:**
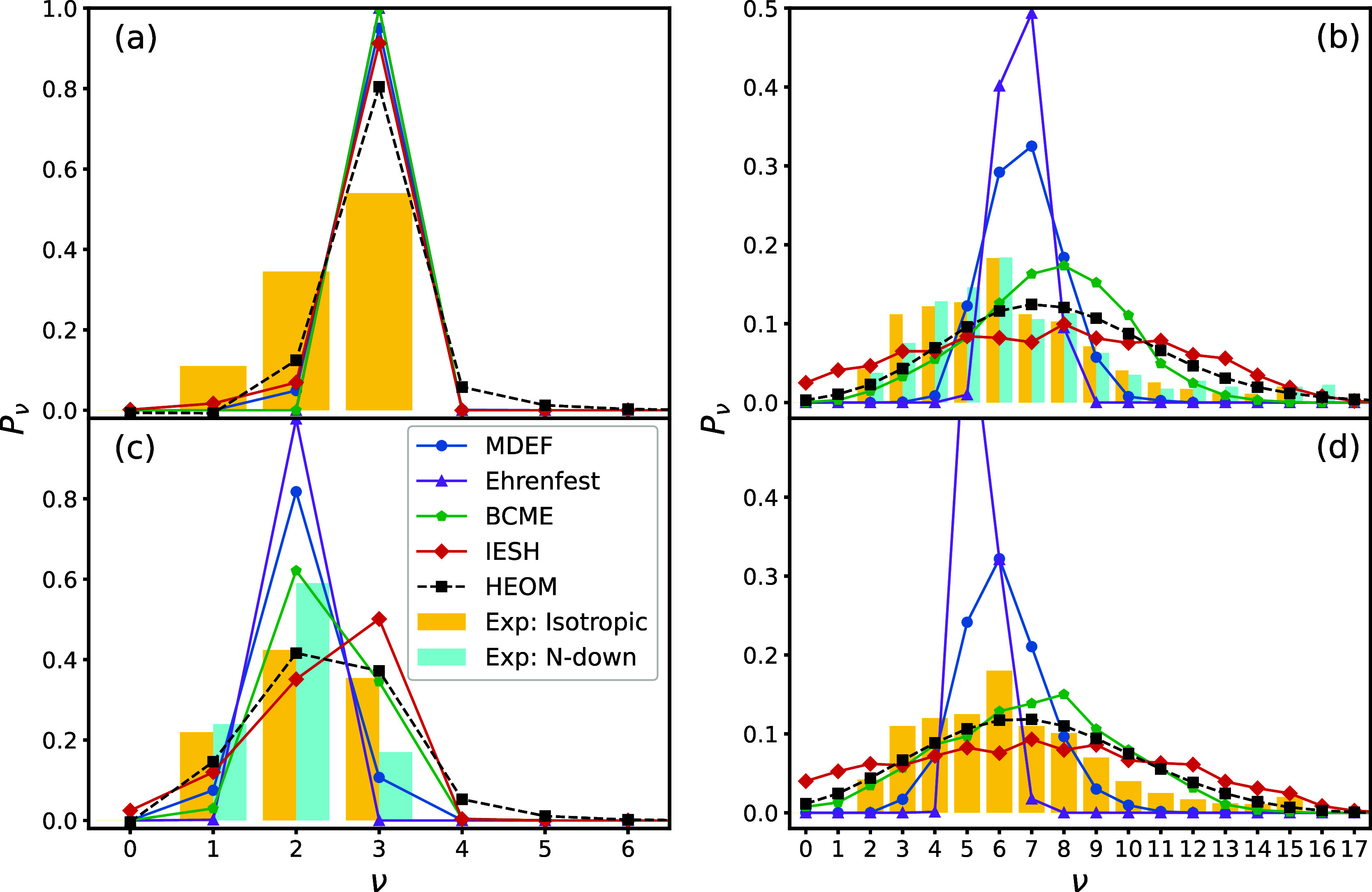
Population of bond vibrational states after scattering. The left column corresponds to ν_ini_ = 3 with (a) KE_ini_ = 0.5 eV and (c) KE_ini_ = 1.0 eV. The right column corresponds to ν_ini_ = 16 with (b) KE_ini_ = 0.5 eV and (d) KE_ini_ = 1.0 eV. Colored lines show MQC calculations taken from ref (42), where we include molecular dynamics with electronic friction (MDEF), Ehrenfest dynamics, broadened classical master equations (BCME), and independent electron surface hopping (IESH). The isotropic experimental data are taken from ref (40). The N-down experimental data for ν_ini_ = 3 and KE_ini_ = 0.95 eV are taken from ref (24), while the N-down experimental data for ν_ini_ = 16 and KE_ini_ = 0.52 eV are taken from ref (69). All experimental data have been normalized.

As a qualitative point of reference, we additionally include experimental data corresponding either to an isotropic initial orientation of the incoming molecule or to cases where the incoming molecule has the N nucleus oriented down toward the surface, the latter serving as a more reliable reference for the model under consideration. By virtue of being numerically exact, a comparison of the HEOM data with experimental results exposes the limitations associated with considering a reduced model of the scattering molecule and neglecting phonons in the surface. For example, consider Figure 4c, which compares the final vibrational distributions after scattering when ν_ini_ = 3 and KE_ini_ = 1.0 eV. The population of the ν = 3 vibrational quantum state is higher, and the population of the ν = 2 state is lower in the HEOM compared to the N-down experiment. This is likely due to the neglect of additional energy dissipation channels, such as energy transfer to phonons in the surface, or nuclear degrees of freedom beyond 2D such as the rotational motion of the molecule. This also emphasizes the importance of scrutinizing approximate dynamics methods of propagation with respect to numerically exact data for the same model, rather than directly comparing against experimental data. Having said that, we find that the experimental data are qualitatively reproduced by HEOM and the employed model in all parameter regimes tested here, particularly for higher vibrational initial states. This confirms that the model captures the essential aspects of nonadiabatic gas-surface scattering and provides a suitable platform to assess approximate MQC methods.

We now proceed with the benchmarking of the MQC methods. In Figure 4a, where ν_ini_ = 3 and KE_ini_ = 0.5 eV, all MQC methods differ from the HEOM result, underpredicting both the relaxation and excitation from the initial vibrational state. In the case of KE_ini_ = 1.0 eV, as shown in Figure 4c, the approaches based on the broadened classical master equation (BCME) and independent electron surface hopping (IESH) best approximate the HEOM data, while molecular dynamics with electronic friction (MDEF) and Ehrenfest dynamics approaches less accurately account for the spread of the vibrational distribution. Such an analysis is also true upon considering an initial vibrational state of ν_ini_ = 16, as shown in Figure 4b for KE_ini_ = 0.5 eV, and Figure 4d for KE_ini_ = 1.0 eV. In both cases, BCME and IESH perform best in comparison to the exact HEOM data, and each becomes more accurate for larger initial kinetic energy of the molecule. Ehrenfest dynamics and MDEF perform comparatively poorly, each greatly underpredicting the spread of the final vibrational distribution despite also predicting a large amount of vibrational relaxation.

**Figure 5:**
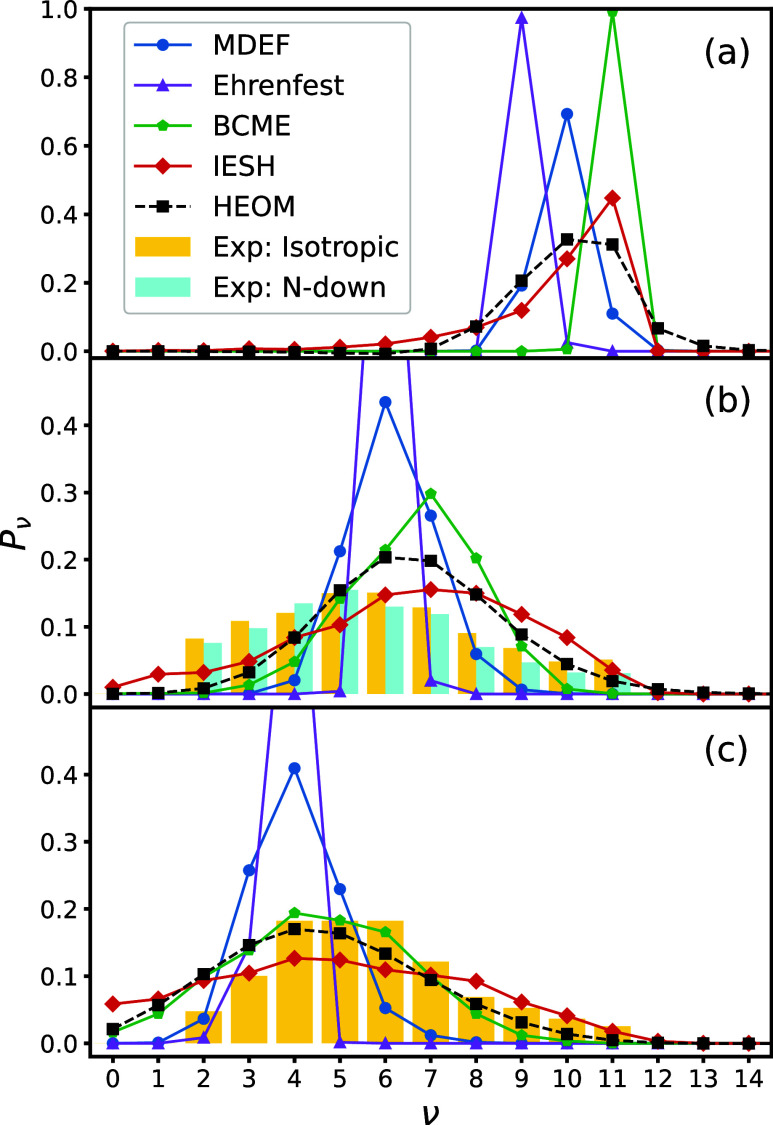
Population of bond vibrational states after scattering for ν_ini_ = 11 for (a) KE_ini_ = 0.2 eV, (b) KE_ini_ = 0.5 eV, and (c) KE_ini_ = 1.0 eV. The isotropic experimental data are taken from ref (40). The N-down experimental data for KE_ini_ = 0.51 eV are taken from ref (69). All experimental data have been normalized.

We further study previously unpublished data of MQC methods in Figure 5a–c, where the final vibrational distribution is shown for an initial vibrational state ν_ini_ = 11 and for initial kinetic energies KE_ini_ ∈ {0.2, 0.5, 1.0 eV}. When KE_ini_ = 0.2 eV, the molecule experiences only a small amount of vibrational relaxation. This is because the molecule requires more energy to reach the crossing between the *U*_0_ and *U*_1_ states, where the interaction with EHPs is strongest. This case is of particular interest as nonadiabatic effects remain prevalent, while quantum nuclear effects become more prominent in the low kinetic energy regime, which should lead to a natural breakdown of the MQC methods. BCME is inaccurate in this regime, predicting no vibrational relaxation at all, while MDEF and Ehrenfest dynamics each predict some relaxation but largely quantitatively differ from the exact result. IESH fairs best out of the methods tested in this regime but is still not a quantitatively accurate representation of the exact data. The BCME and IESH methods each converge toward the exact result upon increasing the initial kinetic energy of the molecule. Meanwhile, the Ehrenfest and MDEF methods prove to be unreliable in all kinetic energy regimes.

To summarize the comparison with the MQC methods, except for the failure of the BCME approach for very low initial kinetic energy, the exact data are approximated to a reasonable degree by both the BCME and IESH approaches. However, BCME slightly underpredicts the spread of the vibrational distribution, and IESH slightly overpredicts the spread of the vibrational final state distribution in most cases. In contrast, Ehrenfest dynamics and MDEF are consistently inaccurate, greatly underestimating the spread of the final distribution. The results presented here demonstrate the inadequacy of a mean-field description of the electronic forces acting on the nuclear vibrations in the scattering molecule, which is central to Ehrenfest dynamics. MDEF, meanwhile, relies on an assumption of weak nonadiabaticity and is thus unreliable in capturing the extent of the nonadiabatic EHP excitation effects that drive the vibrational relaxation of the molecule. BCME and IESH are not reliant on these assumptions and are thus better suited to accurately treat the vibrational relaxation in this scattering system.

They also appear to become more accurate for larger initial kinetic energies of the molecule, where an MQC approximation should be more reliable. This is consistent with previous data, where the BCME approach has been shown to accurately reproduce numerically exact data in classical regimes.^70^ In the lower kinetic energy regime, the molecule remains at the surface longer and has more time to interact with phonons, while directional steering effects and rotational–vibrational coupling are more important. Additionally, the quantum nature of nuclei is more important at lower kinetic energies, meaning that quantum nuclear effects and nonadiabatic effects both contribute strongly to the dynamics. Further development of MQC methods in this regime would be of great value to better understand ultrafast surface dynamics.

The discrepancy between the exact theoretical data and the experimental results, where such comparable experimental results exist, also highlights the need to explore further expansions to the model to capture the rotational–vibrational coupling of the molecule, the orientation of the molecule with respect to the surface, and the phonons in the surface. This is especially true since it has been shown that a significant amount of the vibrational relaxation can be accounted for via an accurate representation of the high-dimensional adiabatic potential energy surface.^71^

## 4 Conclusions

We have presented a theoretical approach to simulate the scattering of molecules from metal surfaces that treats the electronic coupling of the molecule to the surface electrons in a fully quantum mechanical and numerically exact manner via the HEOM approach. The approach is facilitated by a matrix product state representation of the state of the system, enabling the treatment of vibrational basis sets that were previously prohibitively large. As a demonstrative example of the method, we considered the scattering of NO from Au(111), where experiments have shown the dynamics to be strongly dependent on the nonadiabatic electronic coupling to the surface, resulting in multiquantum relaxation in the bond vibrations due to scattering. The HEOM method in conjunction with the model employed qualitatively reproduces the physics observed in experiments and has the potential to serve as a crucial tool for validating underlying scattering models in relation to experimental data.

Additionally, we used our HEOM approach to benchmark a range of mixed quantum-classical approaches, from which we are able to identify the broadened classical master equation and surface hopping methods as the most accurate in capturing the nonadiabatic vibrational relaxation of the bond mode. Ehrenfest dynamics and MDEF perform poorly in comparison. The generally poor performance of all tested MQC methods in the low kinetic energy regime, coupled with the current void in available experimental data, presents an intriguing opportunity for new experimental investigations. Our hope is that the presence of a numerically exact benchmark in the form of HEOM motivates the further development of improved MQC methods that can simulate the dynamics of high-dimensional reactive chemical dynamics at surfaces. The theoretical approach introduced and applied here has the potential to treat higher-dimensional systems and can incorporate a phonon bath in the metal surface, the significance of which we have identified through comparisons with available experimental data. With this in mind, further application of the HEOM method developed here to molecular scattering problems will be the subject of future work.

## Supporting Information Available

Further details of the theory, parameters of the model, details of the computation, and additional results (PDF)
