# Supplementary material for: Nonadiabatic Quantum Dynamics of Molecules Scattering from Metal Surfaces
Source: J Chem Theory Comput. 2025 Jan 28;21(3):1054–63. doi: 10.1021/acs.jctc.4c01586 (PMC11823411; doi:10.1021/acs.jctc.4c01586)
Supplement: Supplementary file 1 — ct4c01586_si_001.pdf [file ct4c01586_si_001.pdf]

Supplementary Material for: Nonadiabatic Quantum Dynamics of Molecules Scattering from Metal Surfaces

Riley J. Preston[1*], Yaling Ke[2], Samuel L. Rudge[1], Nils Hertl[3,4], Raffaele Borrelli[5], Reinhard J. Maurer[3,4], Michael Thoss[1]

[1] *Institute of Physics, University of Freiburg, Hermann-Herder-Strasse 3, 79104 Freiburg, Germany*

[2] *Department of Chemistry and Applied Biosciences, ETH Zürich, 8093 Zürich, Switzerland*

[3] *Department of Chemistry, University of Warwick, Gibbet Hill Road, CV4 7AL, Coventry, United Kingdom*

[4] *Department of Physics, University of Warwick, Gibbet Hill Road, CV4 7AL, Coventry, United Kingdom*

[5] *DISAFA, University of Torino, I-10095 Grugliasco, Italy*

* *Email: riley.preston@physik.uni-freiburg.de*

1. MODEL PARAMETERS

The NO/Au(111) model parameters are summarized in Table S1.

**Isolated NO Molecule**	
r_0	1.1510 Å
a_0	2.7968 Å^{-1}
D_0	6.610 eV
**Electronic Coupling to the Surface**	
Γ	1.5 eV
V	$(\Gamma/2\pi)^{1/2}$
$\tilde{a}$	10 Å
**NO/Au Potentials**	
b_0	1.9535 Å^{-1}
z_0	-0.26876 Å
c_0	6.5713 eV
a_1	2.5194 Å^{-1}
r_1	1.2950 Å
D_1	4.1528 eV
a_2	1.0015 Å^{-1}
z_1	1.2350 Å
D_2	2.4171 eV
c_1	8.9587 eV
**Surface**	
T	300 K
μ	0 eV
W	50 eV

TABLE S1: Parameters for the NO/Au model.

2. COMPUTATIONAL DETAILS

The molecular part of the extended wavepacket, consisting of the electronic degree of freedom and two vibrational degrees of freedom, is initially in a product state with the molecule in the neutral state. The initial wavefunction for the vibrational degrees of freedom is then given by

$$\Psi(t=0) = \Phi(z)\chi(r). \tag{1}$$

The z-component of the initial wavepacket is taken to be a Gaussian wavepacket incident on the surface with an average kinetic energy, $\mathrm{KE_{ini}} = p_{\mathrm{ini}}^2/2m_z$, as given by

$$\Phi(z) = \frac{1}{(2\pi\sigma^2)^{\frac{1}{4}}} \exp\Big[-\frac{(z-z_{\mathrm{ini}})^2}{4\sigma^2} + i\frac{p_{\mathrm{ini}}}{\hbar}(z-z_{\mathrm{ini}})\Big]. \tag{2}$$

The width of the initial wavepacket, $\sigma = \frac{20\ \mathrm{au}}{|p_{\mathrm{ini}}|}$, is motivated by experiment and is taken to be inversely proportional to the initial momentum in the z direction[1]. We take $z_{\mathrm{ini}} = 5$ Å to be consistent with previous simulations of this model[2]. In Eq.(2), the basis is given by a discrete variable representation (DVR) of the coordinate z. For converged results, the z degree of freedom is represented by 340 sine-DVR basis states spanning a grid ranging from $z_0 = 0.53$ Å to $z_N = 6.9$ Å.

The vibrational basis states for the bond degree of freedom are the eigenstates of U_0 in the limit $z \to \infty$, where the νth excited state is described by basis state $|\chi_\nu\rangle$. The population of the νth excited state, P_ν, is calculated according to

$$P_\nu(t) = \mathrm{Tr}\{|\chi_\nu\rangle\langle\chi_\nu|\rho_{\mathrm{mol}}(t)\}. \tag{3}$$

In our simulations, only the 30 lowest energy eigenstates are required for converged results.

The temperature of the surface is taken to be $T = 300$ K with $\mu = 0$ eV, while the bandwidth is set to $W = 50$ eV. Consequently, the bath correlation functions are decomposed according to a barycentric decomposition with 9 poles[3]. In each simulation, the time at which the final distribution of populations of the bond vibrational states is computed is taken to be when the distribution stops changing after scattering.

3. RESULTS

Here, we expand on the results presented in the main text. The energy of the $\nu_{\text{ini}} = 16$ state is very close to the energy of intersection between the charged and uncharged potential energy surfaces when the molecule is close to the surface, as demonstrated in Fig. 1(b) in the main text. As a result, energy exchange processes between the molecule and the electrons in the surface are more accessible in comparison to when the molecule is initialized in a less excited state. This manifests in the dynamics of the time dependent electronic population of the molecule as presented in Fig. S1. In Fig. S1(a), three different initial vibrational states are directly compared, in which the molecule initialized in the $\nu_{\text{ini}} = 16$ state starts to become electronically populated earlier and reaches a higher maximum in comparison to the less excited states. The $\nu_{\text{ini}} = 3$ in particular interacts comparatively weakly with the electronic states in the surface at all times throughout the scattering event. In each case, the molecule once again loses its charge upon scattering away from the surface. The electronic population is also dependent on the incoming kinetic energy of the molecule, as observed in Fig. S1(b). A larger initial kinetic energy results in a higher electronic occupation upon scattering from the surface. This is expected from Fig. 1(a) in the main text, where a larger kinetic energy is required to scale the potential barrier before electron interactions with the surface become relevant.

REFERENCES

[1] K. Golibrzuch, P. R. Shirhatti, J. Altschäffel, I. Rahinov, D. J. Auerbach, A. M. Wodtke, and C. Bartels, "State-to-State Time-of-Flight Measurements of NO Scattering from Au(111): Direct Observation of Translation-to-Vibration Coupling in Electronically Nonadiabatic Energy Transfer," J. Phys. Chem. A **117**, 8750–8760 (2013).

[2] J. Gardner, S. Habershon, and R. J. Maurer, "Assessing Mixed Quantum-Classical Molecular Dynamics Methods for Nonadiabatic Dynamics of Molecules on Metal Surfaces," J. Phys. Chem. C **127**, 15257–15270 (2023).

[3] X. Dan, M. Xu, J. T. Stockburger, J. Ankerhold, and Q. Shi, "Efficient low-temperature simulations for fermionic reservoirs with the hierarchical equations of motion method: Application to the Anderson impurity model," Phys. Rev. B **107**, 195429 (2023).

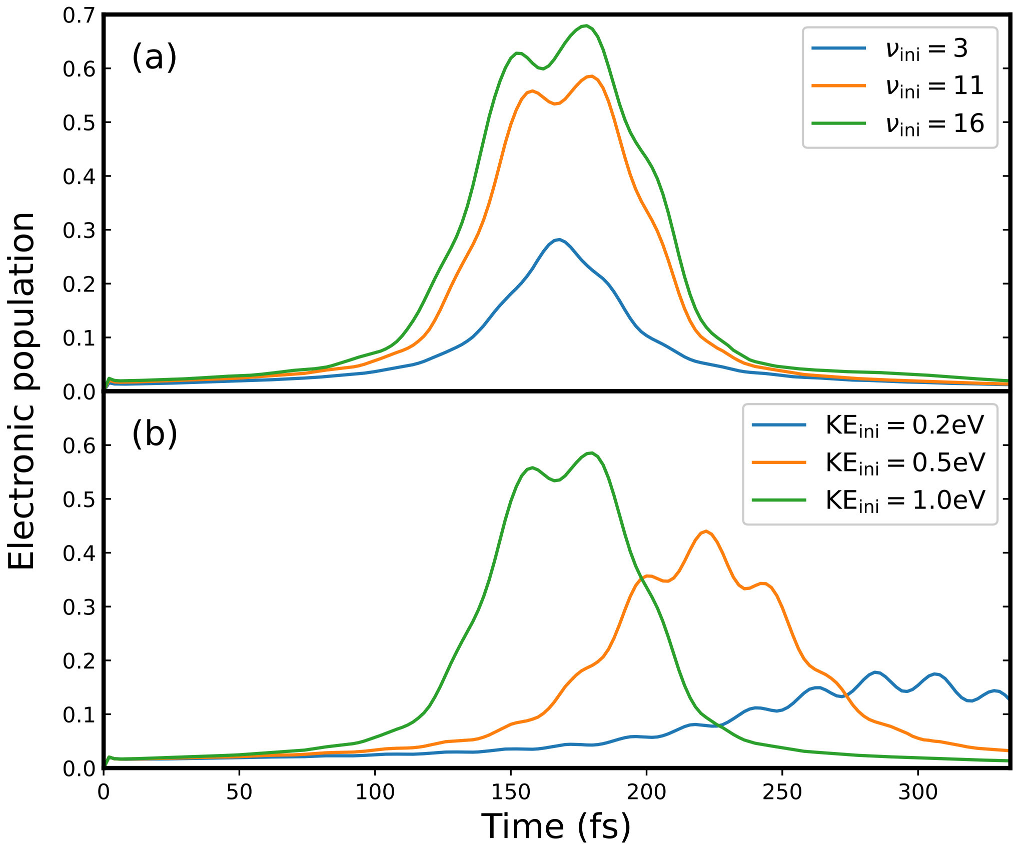

FIG. S1: Time dependence of the electronic population of the scattering molecule: (a) varying ν_{ini} with $\text{KE}_{\text{ini}} = 1.0$ eV, (b) varying KE_{ini} with $\nu_{\text{ini}} = 11$.
